# Modelling the cost-effectiveness of non-invasive prenatal testing in the English sickle cell and thalassaemia screening pathway

**DOI:** 10.1186/s41512-025-00212-9

**Published:** 2026-06-15

**Authors:** Vittoria Vardanega, Ania Bobrowska, Benjamin Ruban-Fell, James A. Doorbar, Silvia Lombardo, Farah Seedat, Amanda Hogan, Mariska M. G. Leeflang, Kathy Mann, Anna Schuh, John Marshall

**Affiliations:** 1 Costello Medical, Cambridge, UK; 2 UK National Screening Committee, Department of Health and Social Care, London, UK; 3 Institute of Infection and Immunity, St George’s University of London, London, UK; 4 NHS Sickle Cell and Thalassaemia Screening Programme, London, UK; 5 Department of Epidemiology and Data Science, Amsterdam University Medical Centers, University of Amsterdam, Amsterdam, The Netherlands; 6 East Genomic Laboratory Hub, Cambridge University Hospitals NHS Foundation Trust, Cambridge, UK; 7 Department of Oncology, University of Oxford, Oxford, UK

**Keywords:** Sickle cell disease, Thalassemia, Screening pathway, Screening, NIPT, Decision tree model, Prenatal, Non-invasive prenatal testing

## Abstract

**Background:**

Sickle cell disease (SCD) and thalassaemia are inherited conditions causing chronic anaemia, increased infection risk and multi-organ failure. Standard of care (SoC) prenatal screening involves carrier blood testing for pregnant women and, if positive, carrier blood testing for biological fathers followed by invasive prenatal diagnosis for pregnancies at risk. Non-invasive prenatal testing (NIPT) presents an alternative pathway which may reduce diagnostic delays and improve equity for pregnant women when the biological father is unavailable by focusing invasive testing exclusively on fetuses shown to have a high risk of SCD by NIPT. This study compares the outcomes of SoC screening with a proposed NIPT pathway replacing the paternal blood testing stage.

**Methods:**

A deterministic decision tree model is used to identify the outcomes of the screening pathways, focusing on the SCD population, from the perspective of the National Health Service (NHS) England. Sensitivity and specificity inputs for NIPT are informed by a separately published minimally acceptable criteria study. Diagnostic outcomes include the number of performed and declined tests and true and false positive/negative diagnoses in each pathway. Economic outcomes include the testing cost of the pathway, the cost per case detected and per accurate diagnosis, and an incremental cost threshold analysis for NIPT. Additional scenario analyses are conducted for the SCD and thalassaemia combined population and for the thalassaemia populations.

**Results:**

When considering an overall cohort of 616,573 pregnancies, implementing the NIPT pathway for the screen-positive SCD population results in an incremental cost of £7,584,551. Of 276 prenatal diagnoses (PND) performed in the SoC arm, 76 show a true positive result for SCD, and 2 false positives are identified. In the NIPT arm, there are 6090 NIPTs and 543 PNDs performed, with 213 true positives and 235 false positives identified. The NIPT pathway costs £33,158 more per case detected, and £368 more per accurate diagnosis than SoC; to obtain no incremental cost per case detected versus the SoC, NIPT would need to cost £45.21.

**Conclusions:**

The presented exploratory analysis may gauge the potential cost-effectiveness of introducing NIPT into the screening pathway, pending further research on the technique’s diagnostic efficacy.

**Supplementary Information:**

The online version contains supplementary material available at 10.1186/s41512-025-00212-9.

## Background

Sickle cell disease (SCD) and thalassaemia are inherited haemoglobin conditions that cause chronic anaemia, increased risk of serious infection, and eventually multi-organ failure [1, 2]. SCD causes episodes of severe pain, chronic organ damage and significantly reduced life expectancy [3]. People with the most severe type of thalassaemia, thalassaemia major, require blood transfusions every 3–5 weeks, causing an excess of iron, which must be managed with chelation therapy to prevent hormonal imbalances and damage to the heart and liver [1, 4, 5]. There are over 17,000 people living with these conditions in the UK [6]. The severity and chronic nature of the conditions highlight the necessity of an effective antenatal screening pathway whereby pregnant women can explore all available options and make an informed decision if the fetus is at risk.

The National Health Service Sickle Cell and Thalassaemia Screening Programme is recommended by the UK National Screening Committee, and offers free testing for pregnant women to determine the risk of a haemoglobin condition to the fetus [7]. All pregnant women living in areas of high SCD prevalence are offered a full blood count and further analyses using high performance liquid chromatography (HPLC) or capillary electrophoresis (CE) techniques [8]. In areas where haemoglobin conditions are less common, full blood count results and a family origin questionnaire are used to determine the need for further analyses. Those deemed to be at potentially high-risk for SCD are offered HPLC/CE to determine their carrier status. In cases where the maternal screening test determines that the pregnant woman is a carrier, the biological father is offered a blood test to determine their carrier status [8, 9]. Subsequently, if the paternal blood test is also positive, or if the biological father test results are not available, the pregnant woman is counselled and offered an invasive test to determine if the fetus is affected [9, 10].

In the current pathway, this test, described as prenatal diagnosis (PND) from here on, is performed either via chorionic villus sampling or amniocentesis [1]. Although studies have shown that these procedures do not result in a statistically significant increase in the rate of miscarriage compared with control groups who have not undergone the procedures, higher risks are often quoted to women [11]. The tests are invasive and may cause discomfort, and many screen-positive women in England decline the offer of PND. The outcome for these pregnancies is then unknown until the newborn is screened [9].

Non-invasive prenatal testing (NIPT) may present a novel approach for the screening pathway. This method analyses cell-free DNA (cfDNA) from the placenta circulating in the maternal bloodstream, which is in most cases representative of the fetal genome, and exploratory studies have suggested this technique may be effective in identifying pregnancies at risk of SCD and thalassaemia [12–14]. NIPT has not yet been proven to be accurate enough for diagnosis of SCD and therefore cannot currently replace PND methods. The usefulness of NIPT, at this stage, may therefore lie in its ability to replace the paternal blood test, whether or not the biological father is available for testing. Women who are SCD carriers could be offered NIPT, which may result in more timely PND. This would also provide more accurate risk information than the materal carrier blood test alone for pregnant women without a paternal carrier blood test [13].

The model described in this manuscript has in part been used to identify the minimally acceptable criteria (MAC) required to inform a diagnostic accuracy study of NIPT, and the results are published in a parallel manuscript [15].

To mitigate for the absence of diagnostic accuracy information for NIPT, the model makes the assumption that in pregnancies which are screen positive for SCD or thalassaemia following NIPT, pregnant women are then offered PND. Early antenatal screening for SCD and thalassaemia maximises the opportunity for pregnant women to make an informed choice [1, 9]. Replacing biological father testing with NIPT in the screening pathway could result in more timely decision making for pregnant women, and would improve equity and risk stratification for the 24% of women with a positive maternal carrier blood test where the biological father is unavailable for blood testing [9, 13]. Eliminating the workload associated with father testing may also improve efficiency within the health service [13].

The main objective of this exploratory model is to estimate the potential impact of introducing NIPT on the National Health Service Sickle Cell and Thalassaemia Screening Programme, in particular with regards to the number of accurate diagnoses in relation to the additional costs by introducing the test, using a decision analytic modelling approach.

## Methods

### Setting

The model used for the current analysis was also used (in part) to identify the MAC required for a diagnostic accuracy study of NIPT, with these results presented in a second manuscript published in parallel [15]. The wider approach to this study is broadly informed by, and performed in line with, the approach proposed by Korevaar et al. (2019) for defining meaningful hypotheses by quantifying the potential consequences of introducing new tests into clinical pathways [16].

A deterministic decision tree model was produced to evaluate the costs and outcomes associated with two SCD and thalassemia antenatal screening test pathways: NIPT and the current standard of care (SoC). The costs and outcomes considered in the base case and scenario analyses were conducted from the perspective of the National Health Service (NHS) Sickle Cell and Thalassaemia Screening Programme in England. The modelled SoC arm involves the maternal carrier blood test stage, followed by the paternal carrier blood test stage; where both the maternal and paternal carrier blood test results are positive, the pregnant woman is offered PND. The intervention considered here, is the introduction of NIPT, modelled as following the maternal carrier blood test stage and in place of the paternal carrier blood test stage before the offer of PND is given to the pregnant woman.

The modelled cohort represents pregnant women undergoing screening in the 2019/2020 English sickle cell and thalassemia pathway. The model initially examined diagnostic outcomes for the following groups:

- Pregnancies affected by sickle cell disease (SCD)
- Pregnancies affected by alpha thalassaemia (caused by genetic mutations in the α-globin gene, which results in decreased synthesis α-globin chains and an imbalance in the α/β-globin chain ratio) [17].
- Pregnancies affected by beta thalassaemia (caused by genetic mutations in the β-globin gene, which results in decreased synthesis β-globin chains and an imbalance in the α/β-globin chain ratio) [18].
- The combined SCD and thalassaemia cohort

Stakeholder engagement included two workshops involving members of the Sickle Cell and Thalassaemia Screening Programme in England, members of the UK National Screening Committee, clinicians, and researchers. These focus group-style workshops were used to obtain the judgement of key experts on the suggested literature inputs to be used in the model, or, in lieu of these data, what would be a reasonable assumption, through informal discussion. Key experts were also involved in discussions outside of these workshops to further refine the model. During the first workshop, the proposed model structure and model inputs were discussed; the feedback received was used to refine the modelling approach and to validate or update the model parameters. During the second workshop the model results were discussed. In the initial workshop, stakeholders noted that NIPT would not currently be diagnostically feasible in a clinical setting within the thalassaemia populations; therefore, the focus of this manuscript is instead on the SCD population, and results for the combined SCD and thalassaemia and individual thalassaemia populations are reported as supplementary material.

### Model structure

**Fig. 1:**
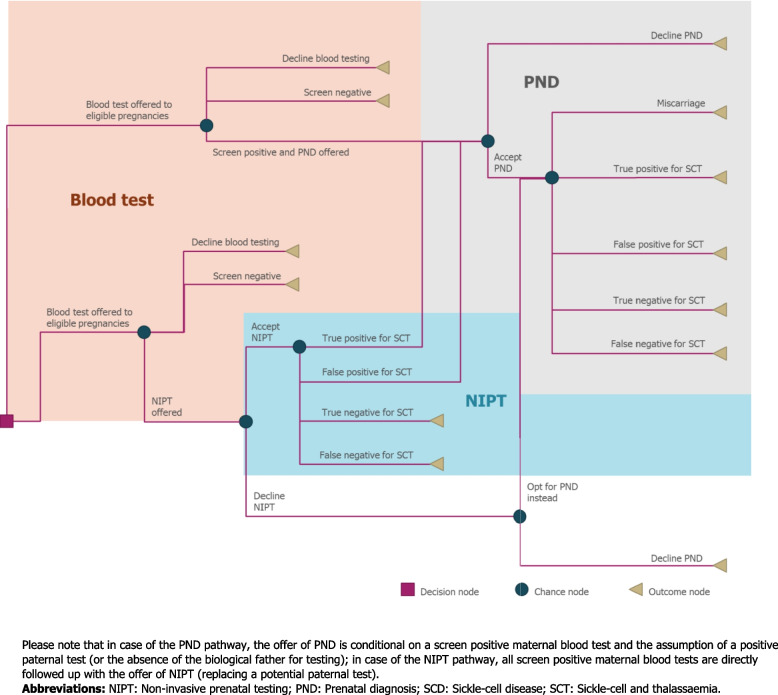
Structure of the decision tree model

The deterministic model is based on a decision tree structure built in Microsoft Excel (Fig. 1). Decision trees are generally appropriate for modelling the short-term outcomes of screening programmes when these outcomes are based on well-defined processes; decision tree structures have further been used in other models of antenatal screening scenarios in a UK population [19–21].

**Fig. 2:**
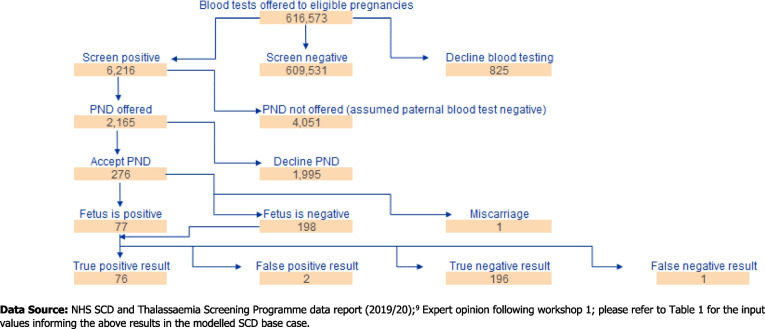
Structure of the PND pathway in the model

**Fig. 3:**
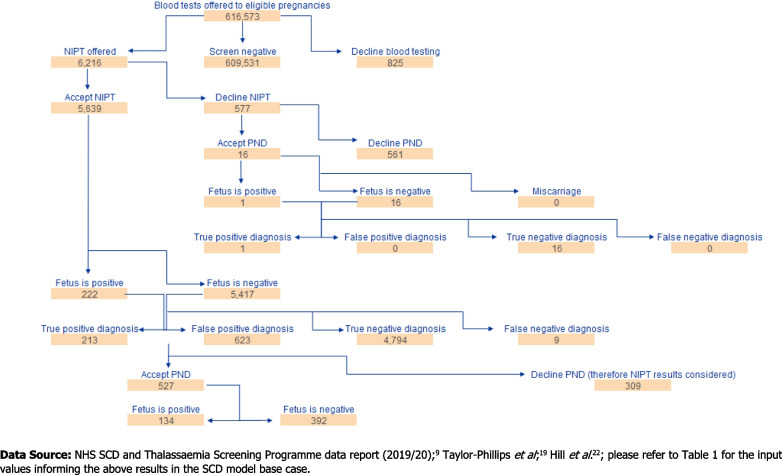
Structure of the NIPT pathway in the model

In both arms, pregnant women enter the decision tree at the point of being offered a blood test as part of the SCD and thalassaemia screening pathway, to indicate whether they are a potential carrier of SCD and/or thalassaemia. Following a screen positive maternal carrier blood test result, pregnant women then progress along the respective modelled screening pathway: to either a paternal carrier blood test in the SoC pathway or NIPT in the NIPT pathway (Figs. 2 and 3, respectively). Women from both pathways found to have pregnancies at high risk of SCD and thalassaemia are offered PND.

While the paternal blood test stage is accounted for in the SoC arm, the results of the paternal test are not explicitly modelled (with regards to the number of positive and negative results). Instead, a proportion of women are assumed to not receive an offer of PND, reflecting where, in a real-world setting in the current screening pathway, the biological father would receive a negative paternal carrier blood test result, indicating that they are not a carrier of SCD and thalassaemia. The remaining women are assumed to have thus received a positive paternal blood test result or that the biological father is not available for testing and are offered PND. In the NIPT pathway, the paternal carrier blood test stage is not modelled, as NIPT would replace this test in the new proposed pathway. Instead, all women who receive a positive maternal carrier blood test result are offered NIPT. Where women receive a positive NIPT result, follow-up PND is then offered, reflecting the current pathway and approach taken in a previously published model on cell-free DNA screening for Downs, Edwards and Patau’s syndrome, which similarly involves an invasive test component [19].

The respective false/true positive or negative test results for each pathway are modelled separately. Across both pathways, the model includes the option for pregnant women to decline testing at any stage of the pathway, at which point pregnant women are considered lost to follow-up, and no outcomes are modelled.

### Model inputs

The full table of inputs for and key assumptions underpinning the model can be seen in Table 1. Epidemiological inputs for the model, such as the number of pregnant women receiving a screen positive result for SCD and/or thalassaemia, were informed by the 2019/2020 SCD and thalassaemia screening report [9]. Inputs on the theoretical uptake of NIPT were obtained from literature through pragmatic searches. Other inputs, such as the likely real-world sensitivity and specificity of PND, or the proportion of negative paternal blood test results, were informed by stakeholder input, gathered in two workshops and through additional engagement throughout the study in line with the approach outlined in Korevaar et al. 2019 [16]. Cost inputs for PND, NIPT and blood testing were obtained from Verhoef et al. 2016, a cost analysis study identified through targeted literature searches [23].

**Table 1:** List of inputs and rationale in the model

Input	Input in Model	Corresponding value in population				Rationale	Source
		SCD and Thal	SCD Base Case	Alpha Thal	Beta hal		
***Blood test***							
Pregnancies offered maternal blood testing	616,573					Number of screening samples for SCD, thalassaemia and other haemoglobin variants in England (2019–2020)	NHS SCD and Thalassaemia Screening Programme data report (2019/2020) [9]
Proportion declining maternal blood testing	0.13%	825				Rate of declines for antenatal screening in England (2019/2020)	NHS SCD and Thalassaemia Screening Programme data report (2019/2020) [9]
Proportion of women who screen positive for SCD and/or thalassaemia (following maternal blood test)	2.13%	13,108	–	–	–	Screen positive results (% of maternal blood test samples, 2019/2020)	NHS SCD and Thalassaemia Screening Programme report (2019/2020) [9]
Pregnancies at risk of SCD (following maternal blood test)	47.42%	–	6216	–	–	Proportion of screen-positive pregnancies (2019/2020)	NHS SCD and Thalassaemia Screening Programme data report, additional information (2019/2020) [9]
Pregnancies at risk of alpha thalassaemia (following maternal blood test)	6.91%	–	–	906	–	Proportion of screen-positive pregnancies (2019/2020)	NHS SCD and Thalassaemia Screening Programme data report, additional information (2019/2020) [9]
Pregnancies at risk of beta thalassaemia (following maternal blood test)	34.45%	–	–	–	4516	Proportion of screen-positive pregnancies (2019/2020)	NHS SCD and Thalassaemia Screening Programme data report, additional information (2019/2020) [9]
***PND***							
Proportion offered PND (as a proportion of all pregnancies offered maternal blood testing)	0.74%	4566	2165	316	1573	Based on number of identified high-risk fathers + number of screen positive pregnancies without father available	Expert opinion following workshop 1
Proportion accepting offer of PND (SCD and Thalassaemia population)	7.88%	360	–	–	–	Number of PND performed for SCD and thalassaemia population divided by number offered (2019/2020)	NHS SCD and Thalassaemia Screening Programme data report (2019/2020) [9]
Proportion accepting offer of PND (SCD population)	12.75%	–	276	–	–	Number of PND performed in SCD population divided by number offered (2019/2020)	NHS SCD and Thalassaemia Screening Programme data report (2019/2020) [9]
Proportion accepting offer of PND (alpha thalassaemia population)	2.22%	–	–	7	–	Number of PND performed in alpha thalassaemia population divided by number offered (2019/2020)	NHS SCD and Thalassaemia Screening Programme data report (2019/2020) [9]
Proportion accepting offer of PND (beta thalassaemia population)	4.07%	–	–	–	64	Number of PND performed in beta thalassaemia population divided by number offered (2019/2020)	NHS SCD and Thalassaemia Screening Programme data report (2019/2020) [9]
Proportion of fetuses positive for SCD and/or thalassaemia (PND-eligible population)	24.85%	89	–	–	–	Proportion of screen positive SCD and/or thalassaemia pregnancies, excluding those with fetal results (2019/2020)	NHS SCD and Thalassaemia Screening Programme data report (2019/20)^1^ excluding fetal carriers
Proportion of fetuses positive for SCD (PND-eligible population)	27.90%	–	77	–	–	Proportion of screen positive SCD pregnancies, excluding those with fetal results (2019/2020)	NHS SCD and Thalassaemia Screening Programme data report (2019/2020) [9] excluding fetal carriers
Proportion of fetuses positive for alpha thalassaemia (PND-eligible population)	14.29%	–	–	1	–	Proportion of screen positive alpha thalassaemia pregnancies, excluding those with fetal results (2019/2020)	NHS SCD and Thalassaemia Screening Programme data report (2019/2020) [9] excluding fetal carriers
Proportion of fetuses positive for beta thalassaemia (PND-eligible population)	12.50%	–	–	–	8	Proportion of screen positive beta thalassaemia pregnancies, excluding those with fetal results (2019/2020)	NHS SCD and Thalassaemia Screening Programme data report (2019/2020) [9] excluding fetal carriers
Sensitivity of PND	99.00%					Experts advised that the sensitivity of PND would be close to 99.00%	Expert advice from workshop 1
Specificity of PND	99.00%					Experts advised that the specificity of PND would be close to 99.00%	Expert advice from workshop 1
***NIPT***							
Proportion accepting NIPT	90.71%	11,891	5639	822	4097	Referenced in Taylor-Phillips 2015 as the assumed proportion who would accept the offer of cfDNA testing following a ‘high-risk’ combined result for T21, T18 and T13 testing in the UK	Taylor-Phillips et al. [19]
Failure rate of NIPT	8.00%	951	451	66	328	Number of NIPT which fail and require retesting	Expert advice obtained during workshop 1
Proportion lost to follow-up (declining NIPT and the alternative offer of PND)	9.29%	1182	561	82	407	Calculated as the proportion who would decline cfDNA testing, using the assumed proportion who would accept the offer of cfDNA testing following a ‘high-risk’ combined result for T21, T18 and T13 testing in the UK	Taylor-Phillips et al. [19]
Proportion accepting PND (following a positive NIPT result)	63.00%	1024	527	83	318	Uptake of invasive testing following a high-risk result	Hill et al. [22]
Proportion choosing PND instead of NIPT	2.86%	35	16	2	12	Calculated as the proportion who decline cfDNA testing in favour of the current standard of care	Taylor-Phillips et al. [19]
Sensitivity of NIPT	96.00%					Calculated based on the minimally acceptable criteria defined in workshop 1	Expert advice obtained during workshop 1; Minimally acceptable criteria parallel publication. [15]
Specificity of NIPT	88.5%						
Costs							
Cost of PND	£1020.00					Testing cost of invasive testing for SCD and thalassaemia	Verhoef et al. [23]
Cost of NIPT	£1203.00					Testing cost of NIPT without phlebotomy	Verhoef et al. [23]

**Abbreviations**: *cfDNA* cell-free DNA, *CI* confidence interval, *NIPT* non-invasive prenatal testing, *PND *prenatal diagnosis, *SCA *sickle cell anaemia, *SCD *sickle cell disease, *Thal* thalassaemia

### Model outcomes

#### Diagnostic outcomes

The model considers the following diagnostic outcomes for the comparison of the SoC and NIPT pathways:

- Number of performed and declined tests in each pathway: PND in the SoC, and NIPT and/or PND in the NIPT pathway
- Number of true and/or false positive and negative diagnoses
- The positive predictive value (PPV), calculated as the number of true positive diagnoses as a proportion of all positive diagnoses
- The negative predictive value (NPV), calculated as the number of true negative diagnoses as a proportion of all negative diagnoses
- The test accuracy, calculated as the number of true positive and true negative diagnoses as a proportion of all diagnoses observed in each pathway.

#### Economic outcomes

The model also considers economic outcomes for each pathway, as detailed below:

- Testing cost, calculated as the total sum of tests conducted in each pathway. In the SoC pathway, the testing cost constitutes the paternal blood tests and PNDs performed. In the NIPT pathway, the testing cost constitutes the NIPTs and PNDs performed. In addition, in the NIPT pathway it is assumed that a fixed percentage of some tests fail and will need to be repeated
- The cost per case detected, calculated as the testing cost for each pathway divided by the number of true positive diagnoses observed
- The cost per accurate diagnosis, calculated as the testing cost for each pathway divided by the combined number of true positive and true negative diagnoses observed

### Threshold analysis

The model includes a series of threshold analyses. These calculate (1) the NIPT cost, (2) the NIPT specificity, and (3) the NIPT sensitivity required for the NIPT pathway to obtain:

- No incremental cost per case detected (defined as £0) vs the SoC pathway
- No incremental cost per accurate diagnosis (defined as £0) vs the SoC pathway

### Model scenarios

The two scenarios used in the model have been justified and discussed in more detail in the parallel publication, but briefly:

- The base case analysis examines the clinical and economic outcomes of the model when a maximum of nine false negative diagnoses are observed in one year, in addition to a PPV of 25%, to match the proportion of pregnancies where both parents are SCD carriers (in situations of a completed testing protocol in the current SoC pathway). See Table 2 for further information
- The scenario analysis examines the clinical and economic outcomes of the model when a maximum of two false negative diagnoses are observed in a year, in addition to a PPV of 25%
- As previously mentioned, it was noted during the initial workshop that NIPT would not currently be diagnostically feasible within the thalassaemia populations; therefore, the thresholds outlined in the scenarios above have been primarily applied to the SCD population in this study

**Table 2:** Assumptions used in the model

Assumption	Justification/Rationale
Screening options are available to all pregnancies regardless of gestational stage	To aid with simplicity of modelling, time-dependency was not included across the model
***SoC pathway***	
Proportion offered PND (following blood test)	Data obtained from the screening programme directly was used to inform the likely number of women who would be offered PND, based on the number of identified high-risk fathers and number of screen positive pregnancies without father available
***NIPT pathway***	
Positive predictive value of completed testing protocol in the current SoC pathway (used as reference for acceptable NIPT outcomes)	Assumed to be 25%, as parents who are both carriers of the single mutation in the β-globin gene which causes SCD have a 1 in 4 chance of producing a child who then inherits both recessive genes. [﻿^1^]
Proportion accepting cfDNA screening in the NIPT pathway	Referenced in Taylor-Phillips et al. that 90.71% would accept the offer of cfDNA testing following a ‘high-risk’ combined result for T21, T18 and T13 testing in the UK [19]

### Scenario analyses

Two scenario analyses were undertaken to identify the impact of varying key inputs on the outcomes of the NIPT pathway. The inputs and variation are as follows:

- Cost of NIPT, varied from a base case value of £1203 (including sample transport and counselling costs as well as the molecular test cost) to £1100 (molecular test cost alone), in line with the cost of the molecular test of NIPT in the SCD setting reported by Verhoef et al. [23]
- Uptake of NIPT, varied from a base case value of 90.71% to 80% and 40%
- Uptake of PND in the NIPT pathway, varied from a base case value of 63% to 30%, 70% and 90%

## Results

### Base case outcomes

The base case analysis examines the wider outcomes of the model when a maximum of nine false negative diagnoses are observed following NIPT and before PND, and the overall PPV is equal to 25% in the NIPT pathway.

Pathway diagrams showing the number of pregnancies at each stage are presented in Figs. 2 and 3 for SoC and NIPT strategies, respectively.

The number of performed tests in the SoC and NIPT strategies are shown in Table 3. In the SoC pathway, a cohort of 616,573 pregnant women are offered the carrier blood test. Of these, 825 decline, 609,531 screen negative, and 6216 screen positive. Of the screen-positive women, 4051 have a negative paternal carrier blood test and do not require further testing, and the other 2165 are offered PND. Two hundred seventy-six PNDs are performed, with 1995 women declining the test.

**Table 3:** Performed and declined tests for the SCD population

Pathway	Number of performed tests		Number of declined tests	
	NIPT	PND	NIPT	PND
SoC	0	276	0	1995
NIPT	6090	543	577	870

In the NIPT pathway, positive NIPT results are followed up by PND

In the NIPT pathway, the cohort of 616,573 pregnant women are offered the carrier blood test. Of these, 825 decline, 609,531 screen negative, and 6216 screen positive and are offered NIPT. There are 5639 NIPTs performed, with 577 women declining; of these 577, 16 accept an alternative offer of PND and 561 decline. Following a positive NIPT result, an additional 527 women accept the offer of PND follow-up (resulting in 543 performed PNDs in total), and 309 decline. As the failure rate of NIPT is 8%, these tests are redone, resulting in a total of 6090 NIPTs performed.

The overall cost for each pathway is shown in Table 4. The SoC pathway is £296,338, compared with an overall cost for the NIPT pathway of £7,880,889 (leading to an incremental cost of £7,584,551 for the NIPT pathway).

**Table 4:** Cost Outcomes for the SCD Population

Pathway	Total	PND	NIPT	Testing cost per case detected	Testing cost per case detected
SoC^a^	£296,337.97	£281,520.00	£0.00	£3901.07	£1,088.34
NIPT	£7,880,889.40	£554,231.55	£7,326,619.64	£37,058.75	£1,456.52
Incremental	+ £7,584,551.43	+ £272,711.55	+ £7,326,619.64	£33,157.68	£368.17

In the NIPT pathway, positive NIPT results are followed up by PND

^a^Testing costs in the SoC pathway also include 4051 paternal blood tests

The diagnostic outcomes for the base case NIPT and PND strategies in the SCD population are shown in Table 5.

**Table 5:** Diagnostic outcomes for the SCD population

Pathway	Number of diagnoses				Lost to follow-up		Accuracy ^a^	PPV	NPV
	True negative	False negative	True positive	False positive	Disease positive	Disease negative			
SoC	196	1	76	2	2	6043	99.00%	97.46%	99.61%
NIPT	5198	10	213	235	0	561	95.67%	47.55%	99.80%

^a^Calculated as number of true (negative + positive) diagnoses divided by the total number of diagnoses. In the NIPT pathway, positive NIPT results are followed up by PND

In the SoC pathway, a PND sensitivity of 99.0% results in a total of 76 true positive and 1 false negative diagnoses. The specificity for this population results in 196 true negative and 2 false positive diagnoses. These outcomes correspond to a PPV of 97.46%, NPV of 99.61% and overall accuracy of 99.00%. In the NIPT pathway, assuming the accuracy for the follow-up PND is the same as in the PND pathway, a NIPT sensitivity of 96.0% results in a total of 213 true positive and 10 false negative diagnoses. The specificity of 88.5% results in 5198 true negative diagnoses, and 235 false positive diagnoses. These outcomes correspond to a PPV of 47.55%, NPV of 99.80% and overall accuracy of 95.67%, for the overall NIPT pathway.

In the SoC pathway, the cost per case detected is £3901, compared with £37,059 for the NIPT pathway, resulting in an incremental cost of £33,158. The cost per accurate diagnosis is £1088 and £1457 for the SoC and NIPT strategies respectively. The NIPT pathway would therefore result in an increased cost of £368 per accurate diagnosis.

The threshold analysis for NIPT in the SCD population base case is shown in Table 6.

**Table 6:** Threshold analysis for NIPT in the SCD population base case

Input	Current Value	Threshold	
		Per case detected	Per accurate diagnosis
Cost of NIPT	£1203.00	£45.21	£875.90
Sensitivity	0.96	N/A	N/A
Specificity	0.89	N/A	N/A

In the NIPT pathway, positive NIPT results are followed up by PND

To achieve no incremental cost per case detected against PND, the cost of NIPT per test performed would need to be £45.21 and could increase up to a maximum of £875.90 to achieve no incremental cost per accurate diagnosis. There is no feasible specificity value for NIPT that would achieve no incremental cost per accurate diagnosis or incremental cost per case detected versus PND, as well as no sensitivity value that would achieve no incremental cost per case detected or per accurate diagnosis versus PND, without also decreasing the initial cost per NIPT performed.

#### Scenario analysis outcomes

The scenario analysis examines the wider outcomes of the model when a maximum of two false negative diagnoses are observed following NIPT and before PND, and the PPV is equal to 25% in the NIPT pathway.

The number of performed and declined tests for the SoC pathway are the same as in the base case, as the SoC inputs are the same.

In the NIPT pathway, the number or performed and declined tests differs from the base case following NIPT. The cohort of 616,573 pregnant women are offered the carrier blood test. Of these, 825 decline, 609,531 screen negative, and 6216 screen positive and are offered NIPT. There are 5,639 NIPTs performed, with 577 women declining; of these 577, 16 accept an alternative offer of PND and 561 decline. Compared to the base case, more women are eligible for PND following a positive NIPT result, resulting in 548 accepting the offer of PND, and 322 declining.

The cost outcomes for the scenario analysis are presented in Table 7.

**Table 7:** Cost Outcomes for the SCD Population Scenario Analysis

Pathway	Total	PND	NIPT	Testing cost per case detected	Testing cost per accurate diagnosis
SoC^a^	£296,337.97	£281,520.00	£0.00	£3901.07	£1088.34
NIPT	£7,902,578.46	£575,920.61	£7,326,619.64	£36,037.96	£1461.49
Incremental	+ £7,606,240.49	+ £294,400.61	+ £7,326,619.64	£32136.89	£373.15

In the NIPT pathway, positive NIPT results are followed up by PND

^a^Testing costs in the SoC pathway also include 4051 paternal blood tests

The outcomes result in a cost per case detected for the PND pathway of £3901 compared to £36,038 for the NIPT pathway (an additional incremental cost per case detected of £32,137 in the NIPT pathway). The cost per accurate diagnosis in the PND pathway results in a cost of £1088, compared to £1461 for the NIPT pathway. The incremental cost per accurate diagnosis between the PND and NIPT strategies would result in an increased incremental cost of £373 for the NIPT pathway.

The diagnostic outcomes for the scenario analysis in the SCD population are shown in Table 8.

**Table 8:** Outcomes for the SCD population scenario analysis

Pathway	Number of diagnoses				Lost to follow-up		Accuracy ^a^	PPV	NPV
	True negative	False negative	True positive	False positive	Disease positive	Disease negative			
SoC	196	1	76	2	2	6043	99.00%	97.46%	99.61%
NIPT	5188	4	219	245	0	561	95.61%	47.25%	99.93%

^a^Calculated as number of true (negative + positive) diagnoses divided by the total number of diagnoses. In the NIPT pathway, positive NIPT results are followed up by PND

The results for the SoC pathway are the same as in the base case, as no SoC inputs are changed in this scenario. In the NIPT pathway, a sensitivity of 99.0% results in a total of 219 true positive and 4 false negative diagnoses. The specificity of 88.0% results in 5188 true negative diagnoses, and 245 false positive diagnoses. These outcomes correspond to a PPV of 47.25%, NPV of 99.93% and overall accuracy of 95.61%.

The threshold analysis for NIPT in the SCD population scenario analysis (MAC of 25% PPV and 2 false negatives) is shown in Table 9.

**Table 9:** Threshold analysis for NIPT in the SCD population scenario analysis

Input	Current value	Threshold	
		Per case detected	Per accurate diagnosis
Cost of NIPT	£1203.00	£45.89	£871.71
Sensitivity	0.99	N/A	N/A
Specificity	0.88	N/A	N/A

*Footnotes*: In the NIPT pathway, positive NIPT results are followed up by PND

To achieve no incremental cost against PND, the cost of NIPT per test performed would need to be £45.89 per case detected, and £871.71 per accurate diagnosis. There is no feasible specificity value for NIPT that would achieve no incremental cost per accurate diagnosis or incremental cost per case detected versus PND, as well as no sensitivity value that would achieve no incremental cost per case detected or per accurate diagnosis versus PND, without also decreasing the initial cost per NIPT performed.

### Sensitivity analyses

Reducing the uptake of NIPT in the NIPT pathway from 90.71 to 80% and 40% results in 5371 and 2685 NIPTs performed, respectively, with an associated testing cost of £6,971,522 and £3,576,548. At 80% uptake, the cost of NIPTs performed is £6,461,271 and the cost of PNDs performed in the NIPT pathway is £510,168; at 40% uptake, the testing cost of NIPTs is £3,230,635, and testing cost of PNDs equates to £345,666.

Altering the uptake of follow-up PND after a positive NIPT result from the base value of 63% to 30%, 70% and 90% results in a total of 267, 602 and 769 PNDs performed. At each increment, the testing cost of the pathway increases to £7,599,389, £7,940,602 and £8,111,208 with the respective testing cost of PND in the pathway as £272,731, £613,944 and £784,550. As the uptake of PND increases, the number of false positives observed in the pathway declines, from 438 at 30% uptake, to 191 at 70%, and 68 at 90%.

Using the cost of NIPT presented in Verhoef et al. (£1100), which is the cost of the molecular test alone (i.e. not including sample transport, counselling and feedback) [23], the testing cost of the NIPT pathway would be £7,253,589, representing a £627,300 decrease in cost compared to the base case cost of NIPT, and £6,957,251 above the testing cost of the SoC pathway. The associated testing cost per case detected and testing cost per accurate diagnosis for the NIPT pathway would subsequently be £34,109 and £1341.

#### Combined SCD and thalassaemia population, alpha, and beta thalassaemia outcomes

The number of performed tests, costs and clinical outcomes for the NIPT and PND strategies in the combined SCD and thalassaemia population, the alpha thalassaemia population, and the beta thalassaemia population are presented in the Supplementary Table section.

For the SCD and thalassaemia population the NIPT pathway has an incremental cost of £16,131,619 compared to the SoC. The incremental cost per case detected is £51,906 and the incremental cost per accurate diagnosis is £328 versus the SoC pathway. The SoC pathway identified 267 true negative, 1 false negative, 88 true positive and 3 false positive diagnoses. The NIPT pathway identified 11,117 true negative, 14 false negative, 293 true positive and 502 false positive diagnoses.

For the alpha thalassemia population, the NIPT pathway has an incremental cost of £1,145,789 compared to the SoC. The incremental cost per case detected is £17,630 and the incremental cost per accurate diagnosis £130 is versus the SoC pathway. The SoC pathway identified 6 true negative, 0 false negative, 1 true positive and 0 false positive diagnoses. The NIPT pathway identified 746 true negative, 2 false negative, 43 true positive and 34 false positive diagnoses.

For the beta thalassaemia population the NIPT pathway has an incremental cost of £5,583,382 compared to the SoC. The incremental cost per case detected is £141,464 and the incremental cost per accurate diagnosis is £240 versus the SoC pathway. The SoC pathway identified 55 true negative, 0 false negative, 8 true positive and 1 false positive diagnoses. The NIPT pathway identified 3,894 true negative, 2 false negative, 37 true positive and 176 false positive diagnoses.

## Discussion

In the model, the pathways diverge following the maternal carrier blood testing stage where 6216 pregnant women screen positive for SCD carrier status. In the SoC pathway, only the 2165 pregnant women without a negative paternal carrier blood test are offered PND and only 276 accept. In the NIPT pathway, in the absence of the paternal carrier blood test, all 6216 maternal carrier pregnancies are offered NIPT, some of which decline the offer and then accept PND, some of which accept NIPT and then, if the test is positive, accept PND as a follow-up test. Because of this, there are more PNDs performed in the NIPT pathway than in the SoC pathway. In part, the cost of these PNDs in addition to the NIPT costs result in an increased cost of £7,584,551 for the NIPT pathway versus the SoC pathway. The modelled NIPT pathway is −3.33% less accurate than the SoC pathway in detecting SCD, though it detected 5002 more true negative diagnosis and 137 more true positive diagnoses than the SoC pathway, however, this should also take into account the 4051 pregnancies identified as not at risk due a negative paternal blood test finding in the SoC pathway. There were 5484 fewer pregnancies considered lost to follow-up, i.e. without a final positive or negative diagnosis, in the NIPT pathway.

The results of this analysis illustrate the complexities of introducing a new screening technology into an established screening pathway. As NIPT is not removing an existing stage in the screening pathway, but is instead modelled as a replacement for the paternal blood test of the current pathway, focusing on modelling the outcomes of the pathway as opposed to the technology alone is a useful approach to determining the impact of NIPT on the outcomes on the overall screening pathway [24]. It should be noted that as the modelled position of NIPT would be prior to the follow-up offer of PND, the number of false positive diagnoses observed following NIPT and resulting PPV can meet a less strict threshold compared to the NPV, as false-positive diagnoses may be identified by the subsequent offer of PND, if accepted. Subsequently, the resulting PPV presented in this study in each scenario may be less desirable than what would be expected in clinical practice—the rationale for this decision has been described in more detail in a parallel publication [15].

The introduction of NIPT in the screening pathway for SCD and thalassaemia, as an alternative to biological father testing, could enhance equity and improve risk stratification for pregnant women when the biological father is unavailable. Consequently, introducing NIPT could have meaningful access, burden, and policy implications for the parts of the population disproportionally affected by SCD and thalassaemia [1, 25–27].

### Threshold analyses

Issues arise when performing cost analyses for novel technologies, particularly in circumstances they have not previously been piloted in, such as for cfDNA screening in the SCD and thalassaemia screening pathway in England. As cost data is scarce for the use of NIPT in this pathway, and a relevant willingness-to-pay threshold is unknown, a full cost-effectiveness or cost-consequence model is unfeasible. This analysis instead takes a threshold approach to the economic evaluation of the two strategies which may have wider applicability in determining the effectiveness of NIPT versus the standard of care from an economic perspective. With this in mind, further research may wish to evaluate the exact threshold, with regards to the cost of NIPT performed, which would be acceptable to the healthcare payer, to better inform future studies of the cost-effectiveness of potential NIPT strategies.

While literature exists on the real-world application of cfDNA screening to the trisomy 21 screening pathway in the United States, there are a range of issues to be considered if this were to be applied to the SCD and thalassaemia screening pathway in England; notably, where the current SoC in the trisomy 21 screening pathway has been demonstrated to be significantly diagnostically inferior when compared with the use of cfDNA testing, the resulting cost outcomes from this literature have limited applicability to other screening pathways [28]. As such, as further research is conducted into the use of NIPT in the SCD and thalassaemia screening pathway, future economic analyses should strive to use cost inputs sourced from real-world studies of sickle cell and thalassaemia screening, where possible, in order to provide the real-world economic implications of introducing this technology.

### Sensitivity analyses

Whilst the main analyses have been performed deterministically, due to the lack of quantifiable information on the degree of uncertainty associated with the majority of model parameters and considering the exploratory nature of the analysis, selected scenario analyses intend to vary some of the key inputs of the model which may differ most in a real-world scenario. While the input for the uptake of NIPT is sourced from the literature, albeit in the context of the trisomy screening pathway in the UK, there is a level of uncertainty as to whether a similar uptake would be observed in the SCD and thalassaemia pathway [19]. Reducing the uptake of NIPT most notably impacts on the overall cost of the pathway, presenting a lower cost as fewer NIPT and follow-up PNDs are performed, and more women are lost to follow-up.

While it is assumed that the uptake of invasive screening following NIPT in this study may be low, the potential real-world uptake is unknown. In this analysis, increasing the uptake of follow-up PND has only marginal impacts on the testing cost of the pathway, but results in a large reduction in the number of false positives observed in the pathway. As such, the low PPV of NIPT may result in a higher uptake of follow-up PND in a real-world context than is modelled in this study, presenting an opportunity for further research.

The cost input from the Verhoef et al. paper represents the value of the molecular test for NIPT, counselling and feedback, and sample transport, without including costs for phlebotomy [23]. This produces an incremental testing cost for the NIPT pathway which is dramatically above the SoC. This again raises key points about the societal willingness-to-pay with a new technology such as NIPT, in particular when there are potential qualitative gains such as the non-invasive nature of the test.

## Strengths and limitations

The methodology underpinning the model used in this study is based on the existing SCD and thalassaemia screening pathway in England, giving a real-world dimension to the model, and designed with consideration to other, similar, screening models, such as in the Warwick trisomy 21 (T21), T18 and T13 screening model [19]. Continuous engagement with stakeholders guided the development of the model, validating both the model inputs before the analysis was conducted, and the results once obtained, adding strength and robustness to the study conclusions.

A key limitation is that where there was a lack of clinical data available on the use of NIPT in the SCD and thalassaemia screening pathway, inputs from literature and stakeholder guidance were used in their stead. Where literature inputs were used, these were informed by a pragmatic literature search, rather than through a full systematic literature review, as was instead the approach taken by the Warwick model [19]. Inputs from stakeholders, in particular on the failure rate of NIPT, are used with the caveat that the actual failure rate in a real-world setting would be unknown, as this would be influenced by the gestational age and body mass index (BMI) of the pregnant woman, the proficiency of the test operator, and quality of the cfDNA sample taken. A further limitation is that the economic outcomes for each pathway in the model are limited to testing costs, with no consideration of the associated downstream costs (i.e. costs associated with care following diagnosis). While it will be important to compare the downstream costs associated with SoC and NIPT, this is beyond the scope of this study, which aimed to estimate the potential impact of introducing NIPT on the performance of the National Health Service Sickle Cell and Thalassaemia Screening Programme.

It is noted that there are some key assumptions used in the model which may limit the applicability of the model to real-world practice. In assuming no time dependency in the model (i.e. all screening options available to all pregnancies regardless of gestational stage), the number of women who undertake NIPT is likely to be higher in the modelled population than would likely be observed in a real-world scenario. This is because data has shown that pregnant women are less likely to accept SCD and thalassaemia screening as the pregnancy develops, although this change in screening uptake as the pregnancy progresses may be negated by the non-invasive nature of NIPT versus the current SoC [9]. Furthermore, inputs sourced from published literature, such as the assumption referenced from Taylor-Phillips et al. that 90.7% of women will accept the offer of a non-invasive form of screening over an invasive method, highlight areas for future large-scale research in real-world conditions [19].

## Conclusion

In conclusion, the results of this study give insight into the effect that replacing biological father blood testing with NIPT may have on the number of diagnoses, overall costs, cost per case detected and cost per accurate diagnosis in the SCD screening pathway. The NIPT pathway accrued higher costs than the SoC but was able to diagnose more pregnancies as positive or negative for SCD rather than losing them to follow-up. Due to the exploratory nature of this analysis and the uncertainty associated with the model inputs, the cost-effectiveness of the NIPT pathway versus the SoC pathway cannot be definitively stated. Aside from more accurate cost information, investigation into the diagnostic accuracy of NIPT is needed to derive high-quality clinical evidence. These data may then be used to populate the model, allowing for a more robust cost-effectiveness analysis to inform discussions about introducing NIPT into the SCD screening pathway in England.

## Supplementary Information

Additional file 1.

Additional file 2: Supplementary Table S1. Number of Performed and Declined Tests for the Combined SCD and Thalassaemia Population. Supplementary Table S2. Cost Outcomes for the Combined SCD and Thalassaemia Population. Supplementary Table S3. Outcomes for the Combined SCD and Thalassaemia Population. Supplementary Table S4. Threshold Analysis for NIPT in the Combined SCD and Thalassaemia Population. Supplementary Table S5. Number of Performed and Declined Tests for the Alpha Thalassaemia Population. Supplementary Table S6. Cost Outcomes for the Alpha Thalassaemia Population. Supplementary Table S7. Outcomes for the Alpha Thalassaemia Population. Supplementary Table S8. Threshold Analysis for NIPT in the Alpha Thalassaemia Population. Supplementary Table S9. Number of Performed and Declined Tests for the Beta Thalassaemia Population. Supplementary Table S10. Cost Outcomes for the Beta Thalassaemia Population. Supplementary Table S11. Outcomes for the Beta Thalassaemia Population. Supplementary Table S12. Threshold Analysis for NIPT in the Beta Thalassaemia Population.

## Data Availability

The Consolidated Health Economic Evaluation Reporting Standard (CHEERS) 2022 has been completed as part of developing this publication and is included as supplementary material (Additional file 1). A copy of the model file can be made available upon reasonable request to Benjamin Ruban-Fell (benjamin.ruban-fell@costellomedical.com).

## Abbreviations

BMI: Body mass index
CE: Capillary electrophoresis
cfDNA: Cell-free deoxyribonucleic acid
HPLC: High performance liquid chromatography
MAC: Minimally acceptable criteria
NIPT: Non-invasive prenatal testing
NHS: National Health Service
NPV: Negative predictive value
PND: Prenatal diagnosis
PPV: Positive predictive value
SCA: Sickle cell and anaemia
SCD: Sickle cell disease
SoC: Standard of care
T13: Trisomy 13
T18: Trisomy 18
T21: Trisomy 21
UK: United Kingdom
