# Supplementary material for: Modelling the cost-effectiveness of non-invasive prenatal testing in the English sickle cell and thalassaemia screening pathway
Source: Diagn Progn Res. 2026 Jun 15;10:17. doi: 10.1186/s41512-025-00212-9 (PMC13267422; doi:10.1186/s41512-025-00212-9)
Supplement: Supplementary file 2 — Additional file 2: Supplementary Table S1. Number of Performed and Declined Tests for the Combined SCD and Thalassaemia Population. Supplementary Table S2. Cost Outcomes for the Combined SCD and Thalassaemia Population. Supplementary Table S3. Outcomes for the Combined SCD and Thalassaemia Population. Supplementary Table S4. Threshold Analysis for NIPT in the Combined SCD and Thalassaemia Population. Supplementary Table S5. Number of Performed and Declined Tests for the Alpha Thalassaemia Population. Supplementary Table S6. Cost Outcomes for the Alpha Thalassaemia Population. Supplementary Table S7. Outcomes for the Alpha Thalassaemia Population. Supplementary Table S8. Threshold Analysis for NIPT in the Alpha Thalassaemia Population. Supplementary Table S9. Number of Performed and Declined Tests for the Beta Thalassaemia Population. Supplementary Table S10. Cost Outcomes for the Beta Thalassaemia Population. Supplementary Table S11. Outcomes for the Beta Thalassaemia Population. Supplementary Table S12. Threshold Analysis for NIPT in the Beta Thalassaemia Population. [file 41512_2025_212_MOESM2_ESM.docx]

SUPPLEMENTARY MATERIAL

Supplementary Table 1. Number of Performed and Declined Tests for the Combined SCD and Thalassaemia Population

| Pathway | **Number of performed tests** | | **Number of declined tests** | | |
| --- | --- | --- | --- | --- | --- |
|  | **NIPT** | **PND** | | **NIPT** | **PND** |
| SoC | 0 | 360 | | 0 | 4,206 |
| NIPT | 12,842 | 1,059 | | 1,217 | 1,784 |

**Footnotes:** Positive NIPT results are followed up by PND in the NIPT pathway.

Supplementary Table 2. Cost Outcomes for the Combined SCD and Thalassaemia Population

| **Pathway** | **Total** | **PND** | **NIPT** | **Testing cost per case detected** | **Testing cost per accurate diagnosis** |
| --- | --- | --- | --- | --- | --- |
|  |  |  |  |  |  |
| **SoC** | £397,931.12 | £367,200.00 | £0.00 | £4,520.89 | £1,120.45 |
| **NIPT** | £16,529,550.31 | £1,080,429.62 | £15,449,040.11 | £56,427.00 | £1,448.73 |
| **Incremental** | + £16,131,619.19 | + £713,229.62 | + £15,449,040.11 | £51,906.11 | £328.27 |

**Footnotes:** Positive NIPT results are followed up by PND in the NIPT pathway.

Supplementary Table 3. Outcomes for the Combined SCD and Thalassaemia Population

| Pathway | **Number of diagnoses** | | | | **Lost to follow-up** | | **Accuracy ^a^** | **PPV** | **NPV** |
| --- | --- | --- | --- | --- | --- | --- | --- | --- | --- |
|  | **True negative** | **False negative** | **True positive** | **False positive** | **Disease positive** | **Disease negative** |  |  |  |
| SoC | 267 | 1 | 88 | 3 | 7 | 12,741 | 99.00% | 97.03% | 99.67% |
| NIPT | 11,117 | 14 | 293 | 502 | 1 | 1,182 | 95.68% | 36.87% | 99.87% |

**Footnotes:** ^a^Calculated as number of true (negative + positive) diagnoses divided by the total number of diagnoses. In the NIPT pathway, positive NIPT results are followed up by PND.

Supplementary Table 4. Threshold Analysis for NIPT in the Combined SCD and Thalassaemia Population

| **Input** | **Current Value** | **Threshold** | |  |
| --- | --- | --- | --- | --- |
|  |  | **Per Case Detected** | **Per Accurate Diagnosis** |  |
| **Cost of NIPT** | £1,203.00 | £45.21 | £875.90 |  |
| **Sensitivity** | 0.96 | NA | NA |  |
| **Specificity** | 0.89 | NA | NA |  |

**Footnotes:** Positive NIPT results are followed up by PND in the NIPT pathway.

Supplementary Table 5. Number of Performed and Declined Tests for the Alpha Thalassaemia Population

| Pathway | **Number of performed tests** | | **Number of declined tests** | |
| --- | --- | --- | --- | --- |
|  | **NIPT** | **PND** | **NIPT** | **PND** |
| SoC | 0 | 7 | 0 | 291 |
| NIPT | 887 | 86 | 84 | 131 |

**Footnotes:** Positive NIPT results are followed up by PND in the NIPT pathway.

Supplementary Table 6. Cost Outcomes for the Alpha Thalassaemia Population

| **Pathway** | **Total** | **PND** | **NIPT** | **Testing cost per case detected** | **Testing cost per accurate diagnosis** |
| --- | --- | --- | --- | --- | --- |
|  |  |  |  |  |  |
| **SoC** | £9,222.12 | £7,140.00 | £0.00 | £9,347.99 | £1,335.43 |
| **NIPT** | £1,155,011.19 | £87,467.93 | £1,067,537.69 | £26,976.98 | £1,465.09 |
| **Incremental** | + £1,145,789.07 | + £80,327.93 | + £1,067,537.69 | £17,628.99 | £129.67 |

Footnotes: Positive NIPT results are followed up by PND in the NIPT pathway.

Supplementary Table 7. Outcomes for the Alpha Thalassaemia Population

| Pathway | **Number of diagnoses** | | | | **Lost to follow-up** | | **Accuracy ^a^** | **PPV** | **NPV** |
| --- | --- | --- | --- | --- | --- | --- | --- | --- | --- |
|  | **True negative** | **False negative** | **True positive** | **False positive** | **Disease positive** | **Disease negative** |  |  |  |
| SoC | 6 | 0 | 1 | 0 | 0 | 881 | 99.00% | 94.29% | 99.83% |
| NIPT | 746 | 2 | 43 | 34 | 0 | 82 | 95.67% | 56.00% | 99.72% |

**Footnotes:** ^a^Calculated as number of true (negative + positive) diagnoses divided by the total number of diagnoses. In the NIPT pathway, positive NIPT results are followed up by PND.

Supplementary Table 8. Threshold Analysis for NIPT in the Alpha Thalassaemia Population

| **Input** | **Current Value** | **Threshold** | |  |
| --- | --- | --- | --- | --- |
|  |  | **Per Case Detected** | **Per Accurate Diagnosis** |  |
| **Cost of NIPT** | £1,203.00 | £45.21 | £875.90 |  |
| **Sensitivity** | 0.96 | NA | NA |  |
| **Specificity** | 0.89 | NA | NA |  |

**Footnotes:** Positive NIPT results are followed up by PND in the NIPT pathway.

Supplementary Table 9. Number of Performed and Declined Tests for the Beta Thalassaemia Population

| Pathway | **Number of performed tests** | | **Number of declined tests** | |
| --- | --- | --- | --- | --- |
|  | **NIPT** | **PND** | **NIPT** | **PND** |
| SoC | 0 | 64 | 0 | 1,449 |
| NIPT | 4,425 | 373 | 419 | 620 |

Supplementary Table 10. Cost Outcomes for the Beta Thalassaemia Population

| **Pathway** | **Total** | **PND** | **NIPT** | **Testing cost per case detected** | **Testing cost per accurate diagnosis** |
| --- | --- | --- | --- | --- | --- |
| **SoC** | £75,729.07 | £65,280.00 | £0.00 | £9,595.33 | £1,199.42 |
| **NIPT** | £5,659,110.83 | £336,237.38 | £5,322,845.69 | £151,059.24 | £1,439.50 |
| **Incremental** | + £5,583,381.77 | + £270,957.38 | + £5,322,845.69 | £141,463.91 | £240.08 |

Supplementary Table 11. Outcomes for the Beta Thalassaemia Population

| Pathway | **Number of diagnoses** | | | | **Lost to follow-up** | | **Accuracy ^a^** | **PPV** | **NPV** |
| --- | --- | --- | --- | --- | --- | --- | --- | --- | --- |
|  | **True negative** | **False negative** | **True positive** | **False positive** | **Disease positive** | **Disease negative** |  |  |  |
| SoC | 55 | 0 | 8 | 1 | 0 | 4,392 | 99.00% | 93.40% | 99.86% |
| NIPT | 3,894 | 2 | 37 | 176 | 0 | 407 | 95.68% | 17.57% | 99.95% |

**Footnotes:** ^a^Calculated as number of true (negative + positive) diagnoses divided by the total number of diagnoses. In the NIPT pathway, positive NIPT results are followed up by PND.

Supplementary Table 12. Threshold Analysis for NIPT in the Beta Thalassaemia Population

| **Input** | **Current Value** | **Threshold** | |  |
| --- | --- | --- | --- | --- |
|  |  | **Per Case Detected** | **Per Accurate Diagnosis** |  |
| **Cost of NIPT** | £1,203.00 | £45.21 | £875.90 |  |
| **Sensitivity** | 0.96 | NA | NA |  |
| **Specificity** | 0.89 | NA | NA |  |
